# Heart Rate Variability in Association with Frequent Use of Household Sprays and Scented Products in SAPALDIA

**DOI:** 10.1289/ehp.1104567

**Published:** 2012-04-22

**Authors:** Amar J. Mehta, Martin Adam, Emmanuel Schaffner, Jean-Claude Barthélémy, David Carballo, Jean-Michel Gaspoz, Thierry Rochat, Christian Schindler, Joel Schwartz, Jan-Paul Zock, Nino Künzli, Nicole Probst-Hensch, SAPALDIA Team

**Affiliations:** 1 Swiss Tropical and Public Health Institute, Basel, Switzerland; 2 University of Basel, Basel, Switzerland; 3 Department of Environmental Health, Harvard School of Public Health, Boston, Massachusetts, USA; 4 SNA-EPIS (Système Nerveux Autonome, Epidémiologie, Physiologie, Exercice, Santé) Research Team (EA 4607), Department of Clinical and Exercise Physiology, University Hospital of Saint-Etienne, PRES (Pole Research and Higher Education), Lyon, France; 5 Divisions of Cardiology and Primary Care Medicine, University Hospitals and Faculty of Medicine, Geneva, Switzerland; 6 Centre for Research in Environmental Epidemiology (CREAL), Barcelona, Spain; 7 Hospital del Mar Research Institute (IMIM), Barcelona, Spain; 8 CIBER Epidemiología y Salud Pública (CIBERESP), Spain

**Keywords:** airway irritants, autonomic nervous system, epidemiology, heart rate variability, observational studies

## Abstract

Background: Household cleaning products are associated with adverse respiratory health outcomes, but the cardiovascular health effects are largely unknown.

Objective: We determined if long-term use of household sprays and scented products at home was associated with reduced heart rate variability (HRV), a marker of autonomic cardiac dysfunction.

Methods: We recorded 24-hr electrocardiograms in a cross-sectional survey of 581 Swiss adults, ≥ 50 years of age, who answered a detailed questionnaire regarding their use of household cleaning products in their homes. The adjusted average percent changes in standard deviation of all normal-to-normal intervals in 24 hr (24-hr SDNN) and total power (TP) were estimated in multiple linear regression in association with frequency [< 1, 1–3, or 4–7 days/week, unexposed (reference)] of using cleaning sprays, air freshening sprays, and scented products.

Results: Decreases in 24-hr SDNN and TP were observed with frequent use of all product types, but the strongest reductions were associated with air freshening sprays. Compared with unexposed participants, we found that using air freshening sprays 4–7 days/week was associated with 11% [95% confidence interval (CI): –20%, –2%] and 29% (95% CI: –46%, –8%) decreases in 24-hr SDNN and TP, respectively. Inverse associations of 24-SDNN and TP with increased use of cleaning sprays, air freshening sprays, and scented products were observed mainly in participants with obstructive lung disease (*p* < 0.05 for interactions).

Conclusions: In predominantly older adult women, long-term frequent use of household spray and scented products was associated with reduced HRV, which suggests an increased risk of cardiovascular health hazards. People with preexisting pulmonary conditions may be more susceptible.

The health hazards associated with household cleaning products are a growing public health concern. Although earlier studies identified the use of cleaning products to be a risk factor for work-related asthma among cleaners employed in industrial and domestic settings (Medina-Ramón et al. 2005; Nielsen and Bach 1999; Rosenman et al. 2003; Zock et al. 2001), more recent studies have observed that nonprofessional use of household cleaning products and air fresheners in domestic settings may be a risk factor for developing asthma (Zock et al. 2007) and breast cancer in females (Zota et al. 2010).

The indoor use of household cleaning products and air fresheners, including products with spray application, may result in inhalational exposures to toxic volatile product constituents [e.g., volatile organic compounds (VOCs)], which are emitted during application, and to secondary pollutants that are formed when these primary constituents react with the indoor environment (e.g., with ozone and secondary organic aerosols) (Bello et al. 2010; Singer et al. 2006; Wolkoff et al. 1998). A wide range of adverse health effects has been observed with indoor exposure to VOCs in nonindustrial environments, including mucosal membrane irritation and systemic effects such as fatigue and poor concentration (Bernstein et al. 2008). A recent statement by the American Heart Association (AHA) on air pollution and cardiovascular disease summarized the role of ambient particles, gases, and chemical substances, including VOCs, in the development of cardiovascular disease (Brook et al. 2010). However, it is largely unknown whether indoor aerosol exposures from household cleaning and air freshening products affect cardiovascular health.

The objective of this study was to examine whether long-term nonprofessional use of household cleaning sprays, air freshening sprays, and scented products in domestic settings was associated with reduced heart rate variability (HRV), an established marker of cardiac autonomic dysfunction and increased cardiovascular events and mortality (Dekker et al. 1997; Kleiger et al. 1987; Tsuji et al. 1996), among participants in the Swiss Cohort Study on Air Pollution and Lung and Heart Diseases in Adults (SAPALDIA). SAPALDIA participants who participated in the present study were predominantly women, many of whom were full-time homemakers, which provided a unique opportunity to carry out our objective.

## Methods

*Study population.* SAPALDIA is a multicenter, population-based prospective cohort study consisting of a random sample of 9,561 adults who were 18–60 years of age when they were recruited from eight regions in Switzerland (Martin et al. 1997). The baseline survey was conducted in 1991 when participants were administered medical examinations, including spirometry testing, and a detailed health questionnaire. The second assessment (SAPALDIA 2) of 8,047 study participants (84.2%) was conducted from 2001 to 2003 and also included HRV measurements and special questionnaires on work-related exposures (Ackermann-Liebrich et al. 2005). From these participants who were ≥ 50 years of age at the time of SAPALDIA 2 (*n* = 4,645), 1,846 individuals (955 women, 891 men) were randomly selected for 24-hr electrocardiogram (ECG) monitoring to assess HRV (Felber Dietrich et al. 2006).

In addition, a detailed questionnaire on household cleaning activities was administered to all SAPALDIA 2 participants who responded positively (*n* = 3,255) to the following question from the health questionnaire, “Have you been the person doing the cleaning and/or washing in your home in the last ten years?” This cross-sectional analysis was restricted to 851 individuals ≥ 50 years of age who had valid HRV measurements and who had completed the household cleaning questionnaire [for a flow chart describing participation, see Supplemental Material, Figure 1 (http://dx.doi.org/10.1289/ehp.1104567)]. Of these 851 participants, 188 were excluded for reporting either occupations that used cleaning products at work (*n* = 166) or that involved metalworking, welding, or soldering (*n* = 22). After further exclusion of participants with insufficient exposure or covariate information (*n* = 82), a total of 581 participants contributed to the analyses. The distributions of basic characteristics were similar between the 581 participants included in this analysis and the 808 nonparticipants, who were also ≥ 50 years of age and reported cleaning activities at their homes, but who were not selected for HRV assessment (see Supplemental Material, Table 1).

**Figure 1:**
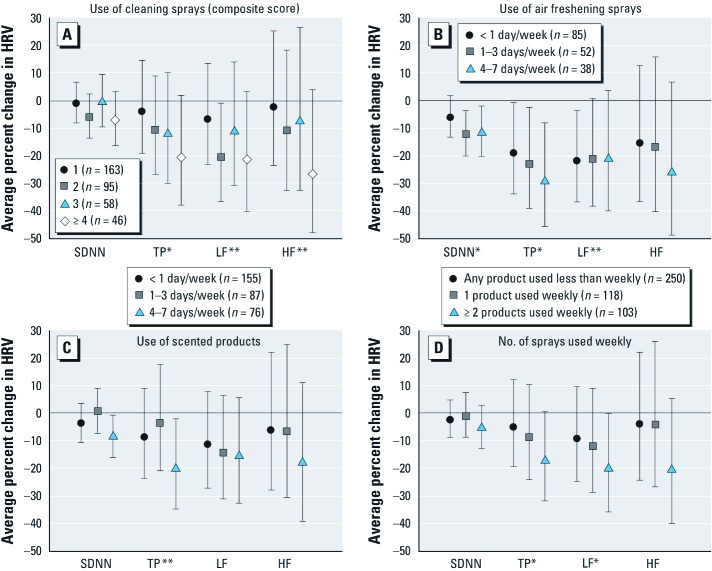
Adjusted average percent change (95% CIs) in 24-hr SDNN, TP, LF, and HF associated with the use of cleaning sprays (*A*), air freshening sprays (*B*), scented products (*C*), and the number of sprays used weekly (*D*). Twenty-four-hour SDNN, TP, LF, and HF were modeled on the logarithmic scale in multiple linear regression as a function of each exposure in separate models and then transformed into average percent change relative to unexposed participants (*n* = 66), after adjusting for sex, age, age^2^, BMI, BMI^2^, alcohol consumption, physical activity, smoking status, environmental tobacco smoke exposure, education, employment status, cardiovascular medication intake, uric acid levels, street and railway noise, traffic-related PM_10_, seasonal effects, and study area. *Ordinal exposure variable p < 0.05. **Ordinal exposure variable p < 0.10.

**Table 1:** Characteristics of participants who reported cleaning in their homes (*n* = 581).

Characteristic	Used spray or scented products (n = 515)	Did not use spray or scented products (n = 66)	p-Value^a^
Age (years) [median (IQR)]		59.8 (54.6, 65.6)		60.4 (56.1, 68.0)		0.38
Male (%)		50 (9.7)		9 (13.6)		0.32
BMI (kg/m2) [median (IQR]		26.0 (22.9, 28.9)		24.6 (22.8, 27.4)		0.12
Smoking status [n (%)]						
Never		272 (52.8)		44 (66.7)		0.04
Former		159 (30.9)		15 (22.7)		0.20
Current		84 (16.3)		7 (10.6)		0.28
ETS exposure (hr/day) [n (%)]						
0		415 (80.6)		56 (84.9)		0.50
< 3		68 (13.2)		7 (10.6)		0.70
≥ 3		32 (6.2)		3 (4.5)		0.79
Alcohol consumption (drinks/day) [n (%)]						
< 1		339 (65.8)		45 (68.2)		0.78
≥ 1		176 (34.2)		21 (31.8)		
Physical activity (hr/week) [n (%)]						
< 0.5		235 (45.7)		34 (51.5)		0.43
0.5 – 2.0		183 (35.5)		17 (25.8)		0.13
> 2.0		97 (18.8)		15 (22.7)		0.51
Uric acid (μmol/L) [median (IQR)]		289 (243, 337)		293 (243, 367)		0.22
Employment status [n (%)]						
Fully/partially employed, in military, or student		76 (14.8)		9 (13.6)		1.00
Unemployed housewife/househusband		218 (42.3)		27 (40.9)		0.89
Retired, sick/disabled, or other		221 (42.9)		30 (44.5)		0.69
Tertiary education levelb [n (%)]						
Low		60 (11.7)		8 (12.1)		0.84
Medium		360 (69.9)		47 (71.2)		0.89
High		95 (18.5)		11 (16.7)		0.87
Taking cardiovascular medication [n (%)]		125 (24.3)		11 (16.7)		0.22
Symptoms and markers of OBSc [n (%)]		212 (54.5)		34 (59.7)		0.32
IQR, interquartile range. ap-Values are based on chi-square and two-sample comparison tests for categorical variables and continuous variables, respectively. bLow, primary school; medium, secondary school/middle school/apprenticeship school; and high, technical college/university. cPercentages represent the 404 exposed and 57 unexposed participants who completed prebronchodilator spirometry and who did not report ever having asthma or taking respiratory medication.						

Ethical approval for the study was given by the central Ethics Committee of the Swiss Academy of Medical Sciences and the Cantonal Ethics Committees for each of the eight examination areas (Aarau, Basel, Davos, Geneva, Lugano, Montana, Payerne, and Wald, Switzerland) and participants signed an informed consent at the examination.

*HRV measurements and analyses.* Holter recordings, described elsewhere by Felber et al. (2006), were made between August 2001 and March 2003. Recorders were placed on participants who had given consent after a detailed health interview. Participants were asked to follow their regular daily routine during the recording period. To avoid a biased result due to methacholine challenge, which was part of the SAPALDIA lung function testing and which, for practical reasons, was performed before the Holter recording, we excluded the first 2 hr of all recordings. The mean ± SD duration of the Holter recordings was 22.4 ± 2.1 hr. The summary measures of HRV were selected as the primary outcomes of interest in this analysis and included the 24-hr value of the SD of all normal RR (NN) intervals (msec 24-hr SDNN), and the following frequency domain variables: total power (TP; ≤ 0.40 msec^2^/Hz), low-frequency (LF) power (0.04–0.15 msec^2^/Hz), and high-frequency (HF) power (0.15–0.40 msec^2^/Hz). The evaluation of SDNN and TP was also limited to nighttime, which was defined as the time when subjects indicated in the diary that they where sleeping (see Felber et al. 2008; Probst-Hensch et al. 2008). To improve normality of the residuals, each HRV parameter was log transformed in this analysis.

*Spirometry testing.* The spirometry protocol was equivalent to that of the European Community Respiratory Health Survey (ECRHS) (Burney et al. 1994). No bronchodilation was applied. Participants performed three to eight forced expiratory lung function maneuvers with the spirometer (model 2200; Sensormedics Yorba Linda, CA, USA), and at least two acceptable measurements of forced vital capacity (FVC) and forced expiratory volume in 1 sec (FEV_1_) were obtained, complying with the American Thoracic Society criteria (American Thoracic Society 1995).

*Respiratory symptoms and medication use.* Presence of asthma was based on positive responses to the questions “Have you ever had asthma?” and, if yes, “Was this confirmed by a doctor?” Shortness of breath was defined as a positive response to the question “Are you troubled by shortness of breath when hurrying on level ground or walking up a slight hill?” Chronic bronchitis was defined as self-reported cough or phlegm during the day or at night on most days for as much as 3 months each year for ≥ 2 years. Medication use for asthma or breathing problems was defined by a positive response to either of the following questions: “Has your doctor ever prescribed medicines, including inhalers, for your breathing?”; “Are you currently taking any medicines, including inhalers, aerosols, or tablets for asthma?”; or “Have you taken medicine for asthma during the last 3 days?”

*Exposure assessment.* The questionnaire module on cleaning and washing in the home, which was adopted from the ECRHS, asked about the frequency of using of 16 different products for domestic cleaning and washing over a period of at least 3 consecutive months since the baseline survey in 1991 (ECRHS 2002). In a previous analysis of Spanish housewives, Medina et al. (2000) compared the frequency responses in this module with a 1-week diary as the gold standard, and the median specificity was 94% across the different cleaning products. We hypothesized that use of products with spray application would better facilitate respiratory exposure to irritants than would nonspray products. Thus, we mainly focused on several spray products used for cleaning glass, furniture, rugs/curtains/carpets, or ovens and on products for ironing, air freshening, and other unspecified purposes. We also examined the use of scented products, which could either be in spray or nonspray form. For each product, the frequency of use was recorded as never, < 1, 1–3, or 4–7 days/week and assigned a score from 0 to 3, respectively. In a preliminary factor analysis, it was determined that the use of cleaning sprays for glass, furniture, and rugs/carpets/curtains contributed to most of the variation in the reported use of spray products in the study sample. A composite score variable for cleaning sprays was subsequently constructed, which was the sum of individual frequency scores for using glass, rug/carpet/curtain, and furniture cleaning sprays with a value ranging from 1 to 9, and divided into four categories (1, 2, 3, ≥ 4). To evaluate the number of sprays used weekly (accounting for all types of sprays, including air freshening sprays), another composite score variable was developed with a value of 1–3 (1, any spray < 1 day/week; 2, 1 spray ≥ 1 day/week; 3, ≥ 2 sprays ≥ 1 day/week).

*Statistical analysis.* Statistical analyses were performed using SAS software (version 9.2; SAS Institute Inc., Cary, NC, USA). Log-transformed 24-hr SDNN, TP, LF, and HF were regressed separately against the different categorical variables of cleaning spray, air freshening spray, scented products, and number of different sprays used weekly in multiple linear regression (PROC GLM). Effect estimates for each exposure frequency category were first expressed as geometric mean ratios, with unexposed participants as the reference group, and then converted into average percent changes. We also evaluated ordinal exposure–response trends by treating exposure variables as continuous, where unexposed participants were assigned a score of zero. Because 24-hr SDNN and TP are in theory mathematically correlated, the Wilks’ lambda test was used to evaluate the overall association between exposure and both outcomes 24-hr SDNN and TP using the MANOVA procedure, which handles multiple correlated outcomes (Scheiner 2001); only *p*-values indicating statistically significant deviation (*p* < 0.05) from the null hypothesis of no association are reported.

All models were adjusted for individual-level covariates that were considered potential confounders of the association between long-term use of household sprays and scented products and HRV including sex (female as reference), age (years), age^2^, body mass index (BMI; kilograms per meter squared), BMI^2^, smoking status [former, current, never (reference)], tertiary education level [high, medium, low (reference)], employment status [retired, sick/disabled, or other; fully/partially employed, in military, or student; unemployed housewife/househusband (reference)], weekly physical activity [to the point of getting out of breath or sweating for < 30 min (reference), between 30 min and 2 hr, or > 2 hr], daily alcohol consumption [≥ 1, < 1 drink (reference)], daily exposure to environmental tobacco smoke [ETS, < 3, ≥ 3, 0 hr (reference)], uric acid concentration measured in serum (micromoles per liter), current cardiovascular medication intake [yes, no (reference)], seasonal effects (based on sine and cosine function of day of examination), street-related noise, train-related noise, average traffic-related particulate matter with aerodynamic diameter < 10 μm (PM_10_) concentration, and study area. The measurement and analysis of personal noise and traffic-related PM_10_ exposures have been described in detail elsewhere (Dratva et al. 2012; Künzli et al. 2009; Liu et al. 2007).

Having ever smoked, obesity (BMI ≥ 30 kg/m^2^), cardiovascular medication intake, and markers or symptoms of obstructive lung disease (OBS) were evaluated as potential effect modifiers. We constructed multiplicative interaction terms between each effect potential modifier and ordinal exposure variables (e.g., exposure scores modeled as continuous variables), and included them in separate multiple linear regression models. Only interactions with *p*-values < 0.05 are reported. In addition, we evaluated interactions with 24-hr SDNN and TP as a combined outcome using the MANOVA procedure described above. OBS was defined as the presence of any of the following markers or symptoms: ratio of forced expiratory volume in 1 sec over forced vital capacity (FEV_1_:FVC) < 0.70, self-reported symptoms of chronic bronchitis, or self-reported shortness of breath. To evaluate effect modification by OBS as distinct from asthma, we excluded all participants who reported an occurrence of asthma or asthma medication intake from the analysis. We did not evaluate self-reported asthma, diabetes, or heart disease for effect modification because of insufficient numbers of observations for statistical comparisons.

*Secondary analyses.* Specific cleaning activities were not recorded in the time activity diaries; thus, we were not able to evaluate the acute effect of household sprays and scented products on HRV. Because HRV during nighttime is less likely to be influenced by short-term disturbances, we estimated adjusted average percent changes of (log-transformed) nighttime SDNN and TP in association with the frequency of use of each product type in multiple linear regression. Linear regression models were also repeated with the reference category for each exposure variable comprising both unexposed participants and those who used the product of interest < 1 day/week.

## Results

Of the 581 participants, 515 reported using any spray or scented product, and 66 reported using neither any spray nor scented product, the latter of whom were considered unexposed in all analyses (Table 1). Both groups were primarily female and were similar with regard to age, BMI, alcohol consumption, employment status, and education level. However, exposed participants included a significantly larger proportion of ever smokers compared with unexposed participants.

Of the 515 exposed participants, 362 reported using cleaning sprays, 175 reported using air freshening sprays, and 318 reported using scented products [see Supplemental Material, Table 2 (http://dx.doi.org/10.1289/ehp.1104567)]. Among participants who used cleaning sprays, 46 were in the highest frequency category (composite score ≥ 4, 12.7%). Approximately 22% and 24% of participants who reported using air freshening sprays and scented products, respectively, used these products 4–7 days/week. The prevalence of current smokers was highest among participants in the most frequent categories for use of cleaning sprays and air freshening sprays and among participants who reported using scented products ≥ 1 day/week. Exposure to ETS ≥ 3 hr/day was highest among participants who used air freshening sprays 4–7 days/week, among those who used scented products ≥ 1 day/week, and among participants with a composite score ≥ 3 for using cleaning sprays. Finally, minimal physical activity (< 0.5 hr/week) was highest in the most frequent categories of all product types.

Unadjusted average percent changes of each summary HRV measure in association with frequency of using cleaning sprays, air freshening sprays, scented products, and number of sprays used weekly are summarized in Supplemental Material, Table 3 (http://dx.doi.org/10.1289/ehp.1104567). Overall, there is a general pattern of reduction in HRV, particularly for TP, with increased usage of all products. The adjusted effect estimates for TP were not considerably different from the corresponding unadjusted estimates, particularly in the highest frequency categories (Figure 1; see also Supplemental Material, Table 4). Decreases in TP were largest for those who used air freshening sprays 1–3 days/week [–23% (95% CI: –39, –2%)] and 4–7 days/week [–29% (95% CI: –46, –8%)] compared with unexposed participants after adjusting for all other covariates. Compared with unexposed participants, similarly large reductions in TP were also observed in the highest frequency categories for use of cleaning sprays and scented products, and number of sprays used weekly, with average decreases in TP ranging between 17–21%. Finally, ordinal trends for lowered TP (*p* < 0.05) were also observed with increased use of cleaning sprays, air freshening sprays, and with the number of sprays used weekly (see Supplemental Material, Table 4).

Compared with unexposed participants, the largest decreases in 24-hr SDNN were associated with using air freshening sprays 1–3 days/week [–12% (95% CI: –20, –4%)] and 4–7 days/week [–11% (95% CI: –20, –2%)] [Figure 1; see also Supplemental Material, Table 4 (http://dx.doi.org/10.1289/ehp.1104567)]. Overall (inverse) associations between both outcomes 24-hr SDNN and TP and using air freshening sprays 1–3 days/week and 4–7 days/week were statistically significant (Wilks’ lambda *p* = 0.02 and *p* = 0.03, respectively). The inverse ordinal trend of the association between air freshening sprays and both outcomes for 24-hr SDNN and for TP was also statistically significant (Wilks’ lambda *p* = 0.02; data not shown). Participants who used scented products 4–7 days/week also had reduced 24-hr SDNN [–9% (95% CI: –16, –1%)] compared with unexposed participants.

Similar to TP, an ordinal trend for decreased LF was observed with the number of sprays used weekly [Figure 1; see also Supplemental Material, Table 4 (http://dx.doi.org/10.1289/ehp.1104567)]. Associations with lower frequency categories of all product types were larger for LF than for HF, but associations with HF were comparable to or larger than associations with LF for the highest frequency categories. Compared with unexposed participants, all discrete comparisons between exposures and LF and HF were not statistically significant with the exception of associations between LF and a composite score of 2 for cleaning spray use, and ≥ 2 sprays used weekly (see Supplemental Material, Table 4).

**Figure 2:**
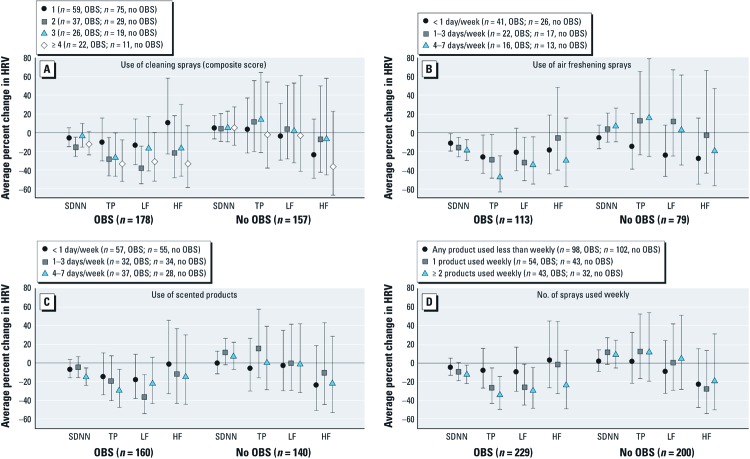
Adjusted average percent changes (95% CIs) in 24-hr SDNN, TP, LF, and HF associated with the use of cleaning sprays (*A*), air freshening sprays (*B*), scented products (*C*), and the number of sprays used weekly (*D*) after stratification by OBS. Twenty-four-hour SDNN, TP, LF, and HF were modeled on the logarithmic scale in multiple linear regression as a function of each exposure in separate models and then transformed into average percent change relative to unexposed participants (*n* = 34, OBS; *n* = 23, no OBS), after adjusting for OBS, sex, age, age^2^, BMI, BMI^2^, alcohol consumption, physical activity, smoking status, environmental tobacco smoke exposure, education, employment status, cardiovascular medication intake, uric acid levels, street and railway noise, traffic-related PM_10_, seasonal effects and study area. Participants who reported doctor-diagnosed asthma or asthma medication use were excluded from this analysis.

We found no major exposure–response differences in 24-hr SDNN and TP for ever smoking and obesity status [see Supplemental Material, Figures 2 and 3 (http://dx.doi.org/10.1289/ehp.1104567)]. However, for all products of interest, negative associations with 24-hr SDNN and TP were observed mainly among participants with markers or symptoms of OBS (Figure 2). Statistically significant interactions between OBS and air freshening sprays, scented products, and the number of spray products used weekly were present for 24-hr SDNN and TP as separate outcomes (all *p* < 0.05) and for 24-hr SDNN and TP as combined outcomes (all Wilks’ lambda *p* < 0.01). The inverse associations of cleaning spray use with 24-hr SDNN and TP were also present mainly among participants with OBS, but a statistically significant interaction was only observed for TP (*p* = 0.10 and *p* = 0.02 for interactions with 24-hr SDNN and TP as separate outcomes, respectively). Associations with air freshening sprays and number of sprays used weekly and LF were also modified so that inverse associations were mainly observed among participants with OBS. Inverse associations between LF and cleaning sprays, air freshening sprays, scented products, and multiple sprays were also larger among participants who reported taking cardiovascular medication (see Supplemental Material, Figure 4), but only for the highest frequency categories of each exposure. A significant interaction was also observed between cardiovascular medication intake and the use of cleaning sprays on both 24-hr SDNN and TP (Wilks’ lambda *p* = 0.03).

*Secondary analyses.* Overall, percent decreases in nighttime SDNN and TP in association with the frequency of household spray and scented product use [see Supplemental Material, Table 5 (http://dx.doi.org/10.1289/ehp.1104567)] were smaller than the percent decreases estimated for the 24-hr period. Decreases in nighttime SDNN in association with use of air freshening sprays 1–3 days/week and 4–7 days/week [–10% (95% CI: –20, 0.6%) and –11% (95% CI: –21, 1.2%), respectively)] were comparable to the average percent changes in 24-hr SDNN.

Overall, the exposure–response patterns were unchanged when the reference category for exposure included both unexposed participants and participants who used products < 1 day/week [see Supplemental Material, Table 6 (http://dx.doi.org/10.1289/ehp.1104567)]. The average percent changes in 24-hr SDNN and TP were not as strongly inverse as the corresponding effect estimates presented in the Supplemental Material, Table 2, where the reference category included unexposed participants only.

## Discussion

Potential health hazards associated with household cleaning products are a growing public health concern, but the effects of regular use on cardiovascular health are largely unknown. In this cross-sectional analysis of predominantly older Swiss women who reported cleaning their own homes, we observed that long-term frequent use of household sprays and scented products was associated with reduced HRV, with the strongest inverse associations observed with air freshening sprays. OBS modified the observed associations, such that participants with either airflow obstruction or self-reported chronic respiratory symptoms (in absence of asthma) appeared to be more susceptible to exposure-associated reductions in HRV than other participants.

Reduced HRV is a marker of cardiac autonomic dysfunction and may increase the risk of all-cause mortality in the general population (Dekker et al. 1997; Tsuji et al. 1996) and in patients with heart failure (Task Force of the European Society of Cardiology 1996), as well as increase the risk of nonfatal cardiovascular events, including myocardial infarction and new-onset hypertension (Singh et al. 1998; Tsuji et al. 1996). Reduced HRV has been described as an intermediate factor between air pollution and cardiovascular morbidity and mortality (Pope et al. 2004; Utell et al. 2002); however, the clinical implications of the associations observed between HRV and the use of household sprays and scented products among older adults are not clear. To our knowledge, this is the first study to evaluate the effect of long-term use of household sprays and scented products on cardiovascular health. The present findings should be verified in other study populations before addressing the clinical implications.

Numerous epidemiologic studies have examined the association between ambient air pollution and HRV, and the general pattern suggests that exposure to particulate matter is associated with increased heart rate and reductions in most indices of HRV among older or other susceptible individuals (Brook et al. 2010), but the biological mechanisms linking ambient air pollution and reduced HRV are not fully understood. The recent AHA statement suggests that inhalation of particulate matter may result in disturbance of the autonomic nervous system balance or heart rhythm by particle interactions with lung receptors or nerves (Brook et al. 2010). We hypothesize a similar mechanism applies to exposures from long-term use of household sprays and scented products, which may result in exposure to VOCs or other toxic air contaminants (Bello et al. 2010; Singer et al. 2006; Wolkoff et al. 1998). Ambient VOCs have been shown to increase the risk of cardiovascular mortality (Theophanides et al. 2007; Tsai et al. 2010), and, in a recent occupational study of healthy young adult females (*n* = 62) working in hair salons, Ma et al. (2010) observed that indoor exposure to nonspecific VOCs was associated with reduced HRV. Mizukoshi et al. (2010) also observed a strong correlation between personal exposure to nonspecific VOCs and a reduction in HRV, particularly HF, in their recent panel study of seven healthy adults who were monitored under usual daily life conditions. Indoor VOCs, particularly the ones from air fresheners, have been shown to interact with ozone to produce secondary organic aerosols indoors. Hence, this may be an additional mechanism of action that could explain stronger effects of air fresheners (Chen and Hopke 2009).

The findings also suggest that those with OBS are more strongly affected by the use of household sprays and scented products, which is of interest. OBS was defined based on symptoms and markers commonly associated with chronic obstructive pulmonary disease (COPD). Although COPD is characterized by chronic airway inflammation, systemic effects have been observed including decreases in HRV and raised systemic inflammation (Sinden and Stockley 2010; Stein et al. 1998; Volterrani et al. 1994). It has been proposed that chronic pulmonary inflammation, by contributing to subclinical systemic inflammation, plays a pivotal role in atherosclerosis and acts as a primary underlying mechanism of cardiovascular morbidity and mortality in association with air pollution exposures (Künzli and Tager 2005). This hypothesis might also apply to effects of long-term exposures to household sprays and scented products. Postbronchodilator spirometry was not performed in this study, so it is possible that some participants classified as having OBS may have had conditions more consistent with asthma (in which airflow obstruction is generally reversible) than with COPD. However, we excluded participants who reported doctor-diagnosed asthma or asthma medication use from the analysis.

Our study has several limitations that should be considered. This was a cross-sectional analysis. Thus, the temporality of exposure–response relations could not be evaluated. The observed findings may also be explained by selection bias should the inclusion of participants in this analysis be associated with both the exposures and outcomes of interest. However, the overall distributions of household spray and scented product use and other characteristics, including smoking and cardiovascular medication intake, were similar between participants and nonparticipants not selected for HRV assessment [see Supplemental Material, Table 1 (http://dx.doi.org/10.1289/ehp.1104567)].

Data collected on the use of household cleaning products were based on self-report, which may result in exposure misclassification. Bias from exposure misclassification is likely nondifferential with respect to HRV, an objective measure, typically leading to a bias towards the null and thus likely resulting in an underestimation of the true association. Exposures resulting from the use of household sprays and scented products also may be modified by home characteristics, such as room size, humidity, ventilation, and temperature, but this information was not collected.

There was also no information available on specific cleaning activities in the time activity diaries recorded during ECG monitoring. Thus, we were not able to estimate acute effects of household sprays and scented products on HRV. It is possible that frequent use of household sprays and scented products over a long duration was associated with an increased likelihood of use of these products immediately before or during ECG monitoring. With the exception of air freshening sprays and nighttime SDNN, average percentage decreases in nighttime HRV in association with exposure were smaller than in corresponding percent decreases in 24-hr HRV, which also raises the question whether principal findings reflect long-term or short-term use of household sprays and scented products.

Although we attempted to control for multiple potential confounders, the observed associations may be biased by residual confounding, such as confounding by sources of indoor air pollution that are known to impair cardiovascular health, including ETS exposures (Barnoya and Glantz 2005) and biomass burning (Baumgartner et al. 2011; McCracken et al. 2011). Indoor measurements of particulate matter and gaseous pollutants were not available in this study, but self-reported information on daily ETS exposure was collected and adjusted for. Additional adjustment for exposure to biomass smoke—which we defined as present if the participant reported use of a wood fireplace, wood burning oven, or either coal, coke, or wood fuel for heating—did not result in any meaningful change in the effect estimates presented (data not shown). Other factors for which we have no available data, such as psychosocial conditions, including anxiety and depression, may also increase the risk of coronary heart disease (Hemingway and Marmot 1999). It is possible that the findings may be explained by unmeasured confounding by these conditions (and other unknown factors) if they increase the risk of reduced HRV and are more prevalent among adults who use household sprays and scented products most frequently. Residual confounding by cigarette smoking, socioeconomic status, and other covariates is also possible. Additional adjustment for cumulative pack-years smoked did not result in any considerable changes in the effect estimates presented (data not shown). Aside from education level, SAPALDIA did not collect information on other proxies for socioeconomic status, such as personal income. Finally, the most frequent users of sprays and scented products tended to be the least physically active on a weekly basis. Although we adjusted for weekly physical activity in our analysis, we cannot exclude the possibility of residual confounding by a hypothetical association between frequent use of sprays and scented products and physical activity on the day of ECG monitoring.

Considering the strength of the observed associations and perceived public health impact, we believe further investigation of the potential effects of exposures to household sprays and scented products on HRV and other cardiovascular outcomes in other study populations is warranted, with emphasis on direct exposure assessment and longitudinal observation of exposures and outcomes. In conclusion, long-term frequent use of household spray and scented products was associated with reduced HRV in a predominantly older population of women, and preexisting pulmonary conditions appeared to increase susceptibility.

## Supplemental Material

(434 KB) PDF
