# Supplementary material for: Heart Rate Variability in Association with Frequent Use of Household Sprays and Scented Products in SAPALDIA
Source: Environ Health Perspect. 2012 Apr 22;120(7):958–64. doi: 10.1289/ehp.1104567 (PMC3404664; doi:10.1289/ehp.1104567)
Supplement: (434 KB) PDF [file ehp.1104567.s001.pdf]

Supplemental Material

Title: Heart Rate Variability in Association with Frequent Use of Household Sprays and Scented Products in SAPALDIA

Authors:
Amar J Mehta, Martin Adam, Emmanuel Schaffner, Jean-Claude Barthélémy , David Carballo, Jean-Michel Gaspoz, Thierry Rochat, Christian Schindler, Joel Schwartz, Jan-Paul Zock, Nino Künzli, Nicole Probst-Hensch, and SAPALDIA-Team

Table of Contents

1. Acknowledgements (page 2)
2. Results (page 3):
- Supplemental Material, Figure 1 (page 3)
- Supplemental Material, Table 1 (page 4)
- Supplemental Material, Table 2 (page 5)
- Supplemental Material, Table 3 (page 6)
- Supplemental Material, Table 4 (page 7)
- Supplemental Material, Figure 2 (page 8)
- Supplemental Material, Figure 3 (page 9)
- Supplemental Material, Figure 4 (page 10)
- Supplemental Material, Table 5 (page 11)
- Supplemental Material, Table 6 (page 12)

ACKNOWLEDGEMENTS

The SAPALDIA team:

Study directorate: T Rochat (p), JM Gaspoz (c), N Künzli (e/exp), LJS Liu (exp), NM Probst Hensch (e/g), C Schindler (s).
Scientific team: U Ackermann-Liebrich (e), JC Barthélémy (c), W Berger (g), R Bettschart (p), A Bircher (a), G Bolognini (p), O Brändli (p), C Brombach (n), M Brutsche (p), L Burdet (p), M Frey (p), U Frey (pd), MW Gerbase (p), D Gold (e/c/p), E de Groot (c), W Karrer (p), R Keller (p), B Knöpfli (p), B Martin (pa), D Miedinger (o), U Neu (exp), L Nicod (p), M Pons (p), F Roche (c), T Rothe (p), E Russi (p), P Schmid-Grendelmeyer (a), M Tamm (P), A Schmidt-Trucksäss (pa), A Turk (p), J Schwartz (e), D. Stolz (p), P Straehl (exp), JM Tschopp (p), A von Eckardstein (cc), JP Zellweger (p), E Zemp Stutz (e).

Scientific team at coordinating centers: M Adam (e/g), E Boes (g), PO Bridevaux (p), D Carballo (c), E Corradi (e), I Curjuric (e), J Dratva (e), A Di Pasquale (s), L Grize (s), D Keidel (s), S Kriemler (pa), A Kumar (g), M Imboden (g), N Maire (s), A Mehta (e), F Meier (e), H Phuleria (exp), E Schaffner (s), GA Thun (g) A Ineichen (exp), M Ragettli (e), M Ritter (exp), T Schikowski (e), G Stern (pd), M Tarantino (s), M Tsai (e), M Wanner (pa)
(a) allergology, (c) cardiology, (cc) clinical chemistry, (e) epidemiology, (exp) exposure, (g) genetic and molecular biology, (m) meteorology, (n) nutrition, (o) occupational health, (p) pneumology, (pa) physical activity, (pd) pediatrics, (s) statistics

Local fieldworkers: Aarau: S Brun, G Giger, M Sperisen, M Stahel, Basel: C Bürli, C Dahler, N Oertli, I Harreh, F Karrer, G Novicic, N Wyttenbacher, Davos: A Saner, P Senn, R Winzeler, Geneva: F Bonfils, B Blicharz, C Landolt, J Rochat, Lugano: S Boccia, E Gehrig, MT Mandia, G Solari, B Viscardi, Montana: AP Bieri, C Darioly, M Maire, Payerne: F Ding, P Danieli A Vonnez, Wald: D Bodmer, E Hochstrasser, R Kunz, C Meier, J Rakic, U Schafroth, A Walder.

Administrative staff: C Gabriel, R Gutknecht.

Supplemental Material, Figure 1 Study design and population

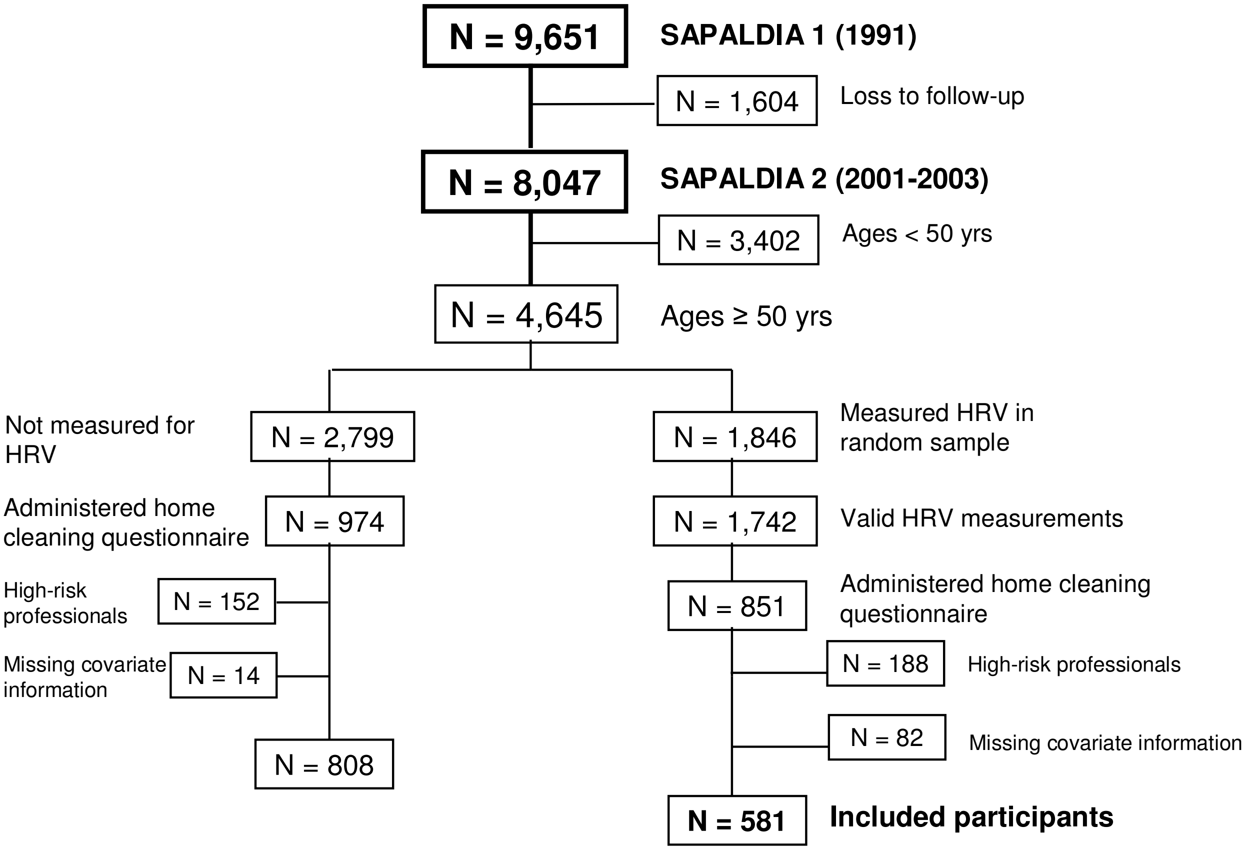

Supplemental Material, Table 1 Comparison of characteristics between selected study participants with heart rate variability measurements and non-selected participants who also reported cleaning activities in their home*

Characteristics	Selected participants (n=581)	Non-selected participants (n=808)
Age, yrs, median (IQR)	59.9 (54.7, 65.8)	60.6 (55.3, 66.8)
Male, (%)	59 (10.2)	89 (11.0)
Body mass index, kg/m^2, median (IQR)	25.8 (22.9, 28.7)	25.5 (22.8, 29.1)
Smoking status, n (%)		
Never	316 (54.4)	425 (52.6)
Former	174 (30.0)	228 (28.2)
Current	91 (15.7)	155 (19.2)
ETS exposure, hours/day, n (%)		
0	471 (81.1)	661 (81.8)
< 3	75 (12.9)	82 (10.2)
≥ 3	35 (6.0)	65 (8.0)
Alcohol consumption, n (%)		
< 1 drink/day	384 (66.1)	513 (63.5)
≥ 1 drink/day	197 (33.9)	295 (36.5)
Physical activity, hrs/week, n (%)		
< 0.5	269 (46.3)	402 (49.8)
0.5 – 2.0	200 (34.4)	241 (29.8)
> 2.0	112 (19.3)	165 (20.4)
Tertiary education level, n (%)		
Low	68 (11.7)	92 (11.4)
Medium	407 (70.1)	593 (73.4)
High	106 (18.2)	123 (15.2)
Taking cardiac medication, n (%)	136 (23.4)	199 (24.6)
Symptoms of chronic obstruction, n (%)#	246 (53.4)	332 (54.3)
Used any spray or scented product, n (%)		
Yes	515 (88.6)	698 (89.9)
No	66 (11.4)	78 (10.1)

* Non-participants who reported cleaning in their home, being equal or older than 50 years of age, and not being in high risk occupations.

# Symptoms of chronic obstruction was defined as having either pre-bronchodilator FEV_1/FVC less than 0.70, or chronic bronchitis, or shortness of breath, *and* in absence of ever having asthma or taking respiratory medication; percentages are expressed relative to the 461 selected and 612 non-participants who completed pre-bronchodilator spirometry and did not report ever having asthma or taking respiratory medication.

Supplemental Material, Table 2 Characteristics of participants who reported on cleaning in their private homes by frequency of using household sprays and scented product (n=581)

	**Unexposed**	**Used cleaning sprays (composite score)**				**Used air freshening sprays (days/week)**			**Used scented products (days/week)**		
**Characteristics**		**1**	**2**	**3**	**≥ 4**	**< 1**	**1-3**	**4-7**	**< 1**	**1-3**	**4-7**
n participants	66	163	95	58	46	85	52	38	155	87	76
Age, yrs, median (IQR)	60.4 (56.1, 68.0)	59.7 (54.1, 65.0)	62.2 (56.9, 66.6)	59.4 (54.0, 65.1)	57.6 (54.5, 67.2)	59.4 (55.1, 65.6)	62.0 (56.2, 65.1)	61.7 (53.5, 65.1)	59.2 (54.0, 64.7)	58.2 (53.7, 63.9)	60.4 (56.1, 68.0)
Male, (%)	9 (13.6)	19 (11.4)	7 (7.4)	5 (8.6)	3 (6.5)	11 (12.9)	2 (3.9)	4 (10.5)	13 (8.4)	8 (9.2)	9 (11.8)
BMI, kg/m2, median (IQR)	24.6 (22.8, 27.4)	25.5 (22.9, 28.9)	26.2 (22.7, 28.4)	27.1 (24.6, 31.1)	26.2 (23.8, 29.2)	26.2 (24.0, 29.1)	26.8 (23.8, 30.7)	27.3 (23.5, 30.5)	26.6 (23.6, 29.4)	25.8 (22.9, 28.1)	24.6 (22.8, 27.4)
Smoking status, n (%)											
Never	44 (66.7)	84 (51.5)	48 (50.5)	29 (50.0)	22 (47.8)	38 (44.7)	29 (55.8)	18 (47.4)	83 (53.6)	44 (50.6)	36 (47.4)
Former	15 (22.7)	54 (33.1)	38 (40.0)	17 (29.3)	10 (21.7)	27 (31.8)	13 (25.0)	10 (26.3)	51 (32.9)	25 (28.7)	25 (32.9)
Current	7 (10.6)	25 (15.3)	9 (9.5)	12 (20.7)	14 (30.4)	20 (23.5)	10 (19.2)	10 (26.3)	21 (13.6)	18 (20.7)	15 (19.7)
ETS exposure, hours/day, n (%)											
0	56 (84.9)	130 (79.8)	81 (85.3)	44 (75.9)	31 (67.4)	69 (81.2)	40 (76.9)	28 (73.7)	133 (85.8)	67 (77.0)	58 (76.3)
< 3	7 (10.6)	23 (14.1)	11 (11.6)	8 (13.8)	11 (23.9)	10 (11.8)	10 (19.2)	7 (18.4)	17 (11.0)	10 (11.5)	11 (14.5)
≥ 3	3 (4.5)	10 (6.1)	3 (3.2)	6 (10.3)	4 (8.7)	6 (7.1)	2 (3.9)	3 (7.9)	5 (3.2)	10 (11.5)	7 (9.2)
Alcohol consumption, n (%)											
< 1 drink/day	45 (68.2)	110 (67.5)	59 (62.1)	44 (75.9)	27 (58.7)	56 (65.9)	33 (63.5)	26 (68.4)	99 (63.9)	55 (63.2)	49 (64.5)
≥ 1 drink/day	21 (31.8)	53 (32.5)	36 (37.9)	14 (24.1)	19 (41.3)	29 (34.1)	19 (36.5)	12 (31.6)	56 (36.1)	32 (36.8)	27 (35.5)
Physical activity, hrs/week, n (%)											
< 0.5	34 (51.5)	80 (49.1)	38 (40.0)	27 (46.7)	28 (60.9)	30 (35.3)	24 (46.2)	25 (65.8)	59 (38.1)	34 (39.1)	46 (60.5)
0.5 – 2.0	17 (25.8)	53 (32.5)	43 (45.3)	22 (37.9)	14 (30.4)	38 (44.7)	18 (34.6)	9 (23.7)	67 (43.2)	30 (34.5)	19 (25.0)
> 2.0	15 (22.7)	30 (18.4)	14 (14.7)	9 (15.5)	4 (8.7)	17 (20.0)	10 (19.2)	4 (10.5)	29 (18.7)	23 (26.4)	11 (14.5)
Uric acid, micromol/l, median (IQR)	293 (243, 367)	295 (243, 348)	287 (232, 335)	293 (265, 336)	278.5 (237, 336)	286 (250, 336)	308.5 (242, 327.5)	287 (245, 336)	281 (235, 343)	290 (239, 337)	293 (243, 367)
Employment status, n (%)											
Full/partially employed, in military, or student	9 (13.6)	21 (12.9)	17 (17.9)	10 (17.2)	8 (17.4)	15 (17.7)	5 (9.6)	8 (21.0)	18 (11.6)	10 (11.5)	13 (17.1)
Housewife/husband	27 (40.9)	64 (39.3)	42 (44.2)	22 (37.9)	24 (52.2)	35 (41.2)	28 (53.9)	16 (42.1)	65 (41.9)	35 (40.2)	31 (40.8)
Retired, unemployed, sick/disabled, or other	30 (44.5)	78 (47.9)	36 (37.9)	26 (44.8)	14 (30.4)	35 (41.2)	19 (36.5)	14 (36.8)	72 (46.4)	42 (48.3)	32 (42.1)
Tertiary education level, n (%)											
Low	8 (12.1)	18 (11.0)	12 (12.6)	8 (13.8)	7 (15.2)	7 (8.2)	8 (15.4)	5 (13.2)	20 (12.9)	9 (10.3)	5 (6.6)
Medium	47 (71.2)	112 (68.7)	72 (75.8)	38 (65.5)	36 (78.3)	63 (74.1)	36 (69.2)	28 (73.7)	102 (65.8)	62 (71.3)	57 (75.0)
High	11 (16.7)	33 (20.3)	11 (11.6)	12 (20.7)	3 (6.5)	15 (17.7)	8 (15.4)	5 (13.2)	33 (21.3)	16 (18.4)	14 (18.4)
Taking cardiovascular medication, n (%)	11 (16.7)	31 (19.0)	29 (30.5)	13 (22.4)	14 (30.4)	24 (28.2)	10 (19.2)	11 (29.0)	40 (25.8)	21 (24.1)	12 (15.8)
Markers and symptoms of obstructive lung disease, n (%)	34 (51.5)	59 (36.2)	37 (38.9)	26 (44.8)	22 (47.8)	41 (48.2)	22 (42.3)	16 (42.1)	57 (36.8)	32 (36.8)	37 (48.7)

Supplemental Material, Table 3 Unadjusted average percent changes* in major indices of HRV in association with frequent use

	24-hour SDNN		Total power		Low frequency		High frequency	
**Exposure variable**	**% change**	**95%CI**	**% change**	**95%CI**	**% change**	**95%CI**	**% change**	**95%CI**
Composite score variable for cleaning sprays								
Unexposed (n=66)		ref		ref		ref		ref
1 (n=163)	-0.3	(-7.6, 7.6)	-3.8	(-19.5, 15.0)	-3.5	(-21.3, 18.2)	2.1	(-20.1, 30.4)
2 (n=95)	-4.6	(-12.3, 3.7)	-8.1	(-24.5, 11.7)	-18.0	(-34.4, 2.6)	-3.9	(-26.5, 25.8)
3 (n=58)	-2.8	(-11.5, 6.7)	-16.0	(-32.5, 4.7)	-10.9	(-30.6, 14.5)	-2.1	(-27.6, 32.5)
≥ 4 (n=46)	-8.9	(-17.5, 0.7)	-20.5	(-37.1, 0.5)	-21.6	(-40.0, 2.5)	-18.9	(-41.2, 11.9)
Air freshening spray (days/week)								
Unexposed (n=66)		ref		ref		ref		ref
<1 (n=85)	-2.8	(-10.1, 5.0)	-12.2	(-27.5, 6.3)	-14.7	(-30.6, 4.9)	-8.0	(-29.2, 19.4)
1-3 (n=52)	-8.0	(-15.8, 0.4)	-19.7	(-35.3, -0.4)	-20.1	(-36.7, 1.0)	-13.4	(-35.6, 16.3)
4-7 (n=38)	-10.7	(-18.9, -1.6)	-20.9	(-37.6, 0.2)	-11.8	(-31.8, 14.0)	-12.0	(-36.4, 21.7)
Scented products (days/week)								
Never (n=66)		ref		ref		ref		ref
<1 (n=155)	-3.7	(-10.4, 3.5)	-6.3	(-21.3, 11.6)	-6.4	(-23.3, 14.2)	-2.9	(-24.7, 25.1)
1-3 (n=87)	1.2	(-6.6, 9.7)	-3.0	(-20.1, 17.8)	-8.6	(-26.7, 13.9)	-0.3	(-24.7, 32.2)
4-7 (n=76)	-9.1	(-16.3, -1.3)	-18.3	(-33.1, -0.3)	-10.6	(-28.8, 12.2)	-10.5	(-33.1, 19.6)
Number of sprays used weekly								
Unexposed (n=66)		ref		ref		ref		ref
Any spray < 1 day/week (n=250)	-2.1	(-9.0, 5.3)	-4.8	(-19.6, 12.7)	-6.9	(-23.3, 13.1)	-2.0	(-22.8, 24.4)
1 spray ≥ 1 day/week (n=118)	-2.6	(-10.2, 5.5)	-12.0	(-27.1, 6.2)	-14.2	(-30.9, 6.4)	-2.0	(-24.8, 27.8)
≥ 2 sprays ≥ 1 day/week (n=103)	-6.6	(-14.1, 1.4)	-17.9	(-32.3, -0.5)	-19.0	(-35.1, 1.0)	-13.1	(-33.8, 14.0)

* 24-hr SDNN , TP, LF, and HF were modeled on the logarithmic scale in multiple linear regression as a function of each exposure (discrete) in separate models and then transformed into average percent change relative to unexposed participants (n=66).

Supplemental Material, Table 4 Adjusted average percent changes* in major indices of HRV in association with frequency use of household spray and scented products

	24-hour SDNN			Total power			Low frequency			High frequency		
**Exposure variable**	**% change**	**95%CI**	**p-value for ordinal trend**	**% change**	**95%CI**	**p-value for ordinal trend**	**% change**	**95%CI**	**p-value for ordinal trend**	**% change**	**95%CI**	**p-value for ordinal trend**
Composite score variable for cleaning sprays												
Unexposed (n=66)		ref			ref			ref			ref	
1 (n=163)	-1.0	(-8.1, 6.6)		-3.8	(-19.3, 14.6)		-6.6	(-23.0, 13.4)		-2.2	(-23.5, 25.0)	
2 (n=95)	-5.9	(-13.6, 2.4)	0.20	-10.7	(-26.9, 9.1)	0.04	-20.6	(-36.4, -1.0)	0.06	-10.5	(-32.4, 18.4)	0.09
3 (n=58)	-0.5	(-9.5, 9.4)		-12.1	(-29.8, 10.0)		-11.1	(-30.7, 14.0)		-7.5	(-32.5, 26.7)	
≥ 4 (n=46)	-7.0	(-16.2, 3.2)		-20.5	(-37.8, 1.7)		-21.3	(-40.1, 3.3)		-26.5	(-47.9, 3.7)	
Air freshening spray (days/week)												
Unexposed (n=66)		ref			ref			ref			ref	
<1 (n=85)	-6.1	(-13.3, 1.8)	0.01	-19.0	(-33.9, -0.7)	0.01	-21.8	(-36.6, -3.6)	0.09	-15.4	(-36.4, 12.6)	0.12
1-3 (n=52)	-12.1	(-19.9, -3.5)		-22.8	(-39.0, -2.3)		-21.0	(-38.0, 0.6)		-16.7	(-40.1, 15.7)	
4-7 (n=38)	-11.4	(-20.2, -1.7)		-29.3	(-45.6, -8.1)		-21.1	(-39.7, 3.4)		-26.0	(-48.8, 6.8)	
Scented products (days/week)												
Never (n=66)		ref			ref			ref			ref	
<1 (n=155)	-3.6	(-10.4, 3.8)	0.12	-8.6	(-23.5, 9.2)	0.07	-11.2	(-27.0, 8.1)		-6.1	(-27.9, 22.3)	0.22
1-3 (n=87)	0.4	(-7.4, 9.0)		-3.4	(-20.7, 17.6)		-14.3	(-31.1, 6.6)		-6.7	(-30.4, 25.1)	
4-7 (n=76)	-8.6	(-15.9, -0.5)		-20.0	(-34.7, -2.0)		-15.6	(-32.6, 5.7)	0.15	-17.9	(-39.3, 11.0)	
Number of sprays used weekly												
Unexposed (n=66)		ref			ref			ref			ref	
Any spray < 1 day/week (n=250)	-2.3	(-8.9, 4.7)	0.33	-4.9	(-19.4, 12.2)	0.04	-9.2	(-24.7, 9.5)	0.04	-3.8	(-24.2, 22.0)	0.10
1 spray ≥ 1 day/week (n=118)	-0.9	(-8.5, 7.2)		-8.6	(-24.2, 10.3)		-11.8	(-28.7, 9.1)		-3.9	(-26.7, 26.0)	
≥ 2 sprays ≥ 1 day/week (n=103)	-5.3	(-12.8, 2.9)		-17.2	(-31.9, 0.7)		-20.0	(-35.9, -0.2)		-20.5	(-40.0, 5.5)	

* Each variable HRV metric was modeled on the logarithmic scale in multiple linear regression as a function of each exposure variable (continuous, or categorical) in separate models, and adjusted for gender, age, age squared, body mass index, body mass index squared, alcohol consumption, physical activity, smoking status, environmental tobacco smoke exposure, education, employment status, cardiac medication, uric acid levels, street and railway noise, seasonal effects and study area.

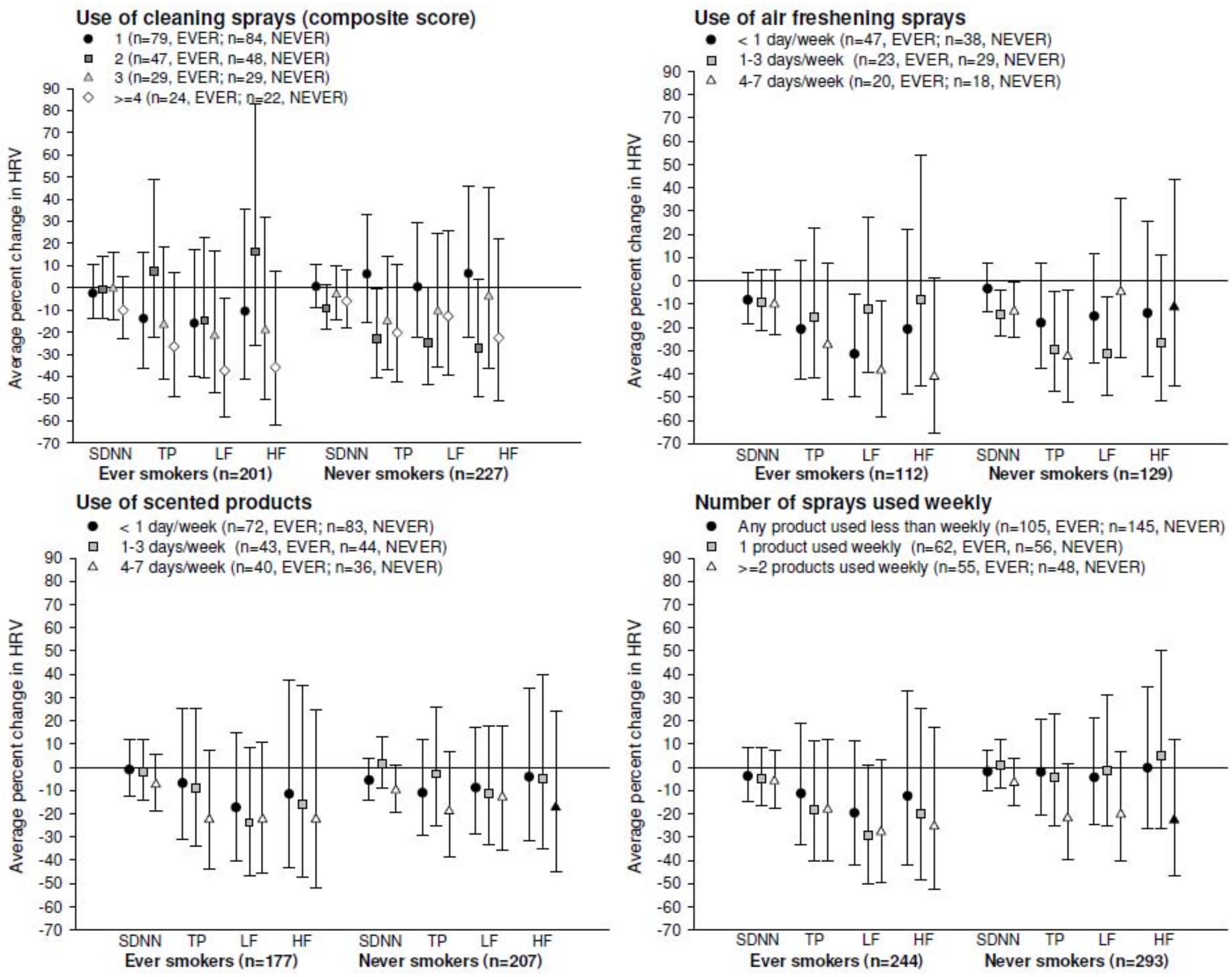

Supplemental Material, Figure 2 Adjusted average percent changes* in 24-hour SDNN, TP, LF, and HF in association with use of cleaning sprays, air freshening sprays, scented products, and with number of sprays used weekly after stratification by smoking status.

* 24-hr SDNN, TP, LF, and HF were modelled on the logarithmic scale in multiple linear regression as a function of each exposure in separate models and then transformed into average percent change relative to unexposed participants (n=22, ever; n=44, never), after adjustment for gender, age, age^2, bmi, bmi^2, alcohol consumption, physical activity, smoking status, environmental tobacco smoke exposure, education, employment status, cardiovascular medication intake, uric acid levels, street and railway noise, traffic-related particulate matter, seasonal effects and study area.

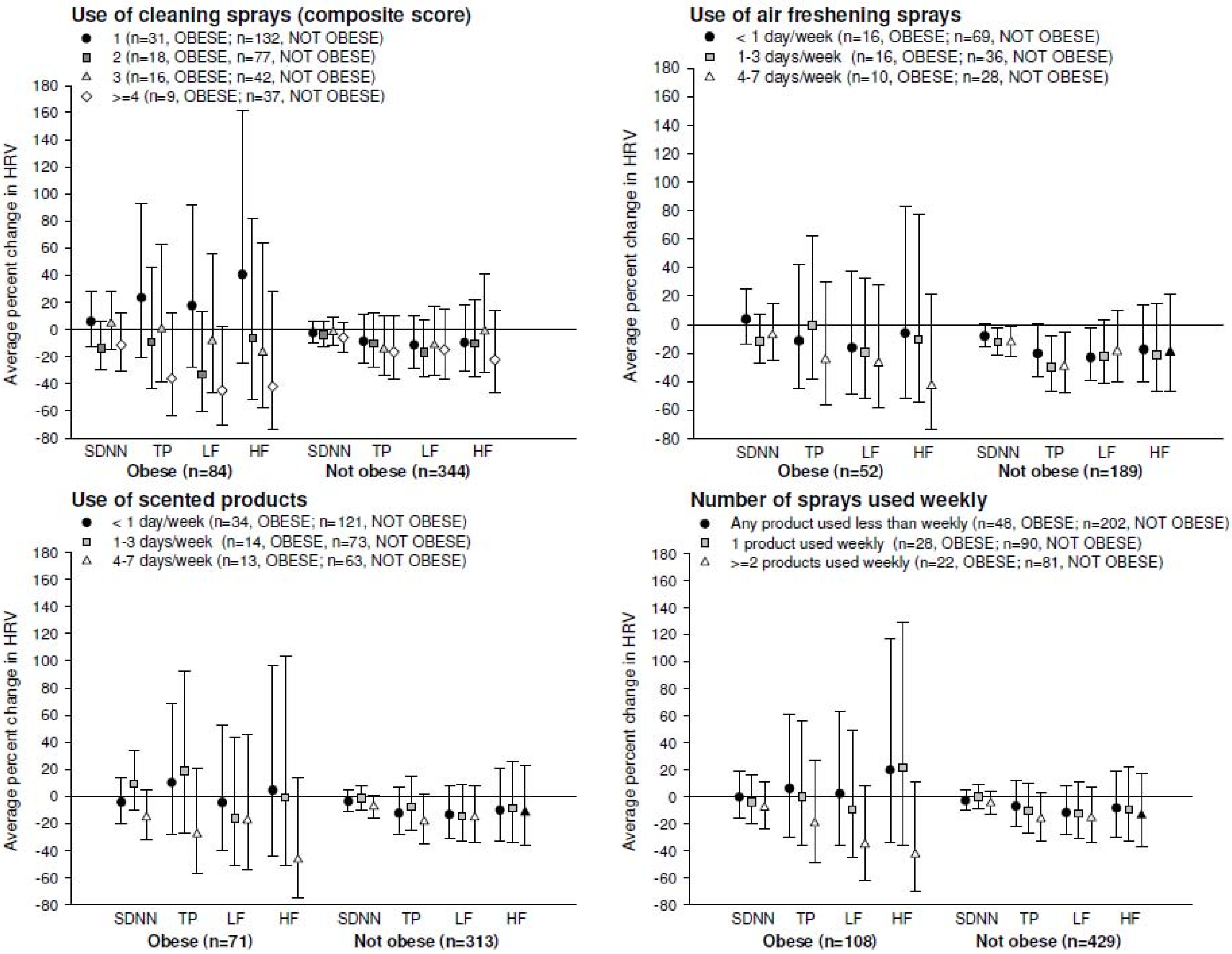

Supplemental Material, Figure 3 Adjusted average percent changes* in 24-hour SDNN, TP, LF, and HF in association with use of cleaning sprays, air freshening sprays, scented products, and with number of sprays used weekly after stratification by obesity (bmi ≥ 30 kg/m^2).

* 24-hr SDNN, TP, LF, and HF were modelled on the logarithmic scale in multiple linear regression as a function of each exposure in separate models and then transformed into average percent change relative to unexposed participants (n=10, obese; n=56, not obese), after adjustment for gender, age, age^2, bmi, bmi^2, alcohol consumption, physical activity, smoking status, environmental tobacco smoke exposure, education, employment status, cardiovascular medication intake, uric acid levels, street and railway noise, traffic-related particulate matter, seasonal effects and study area.

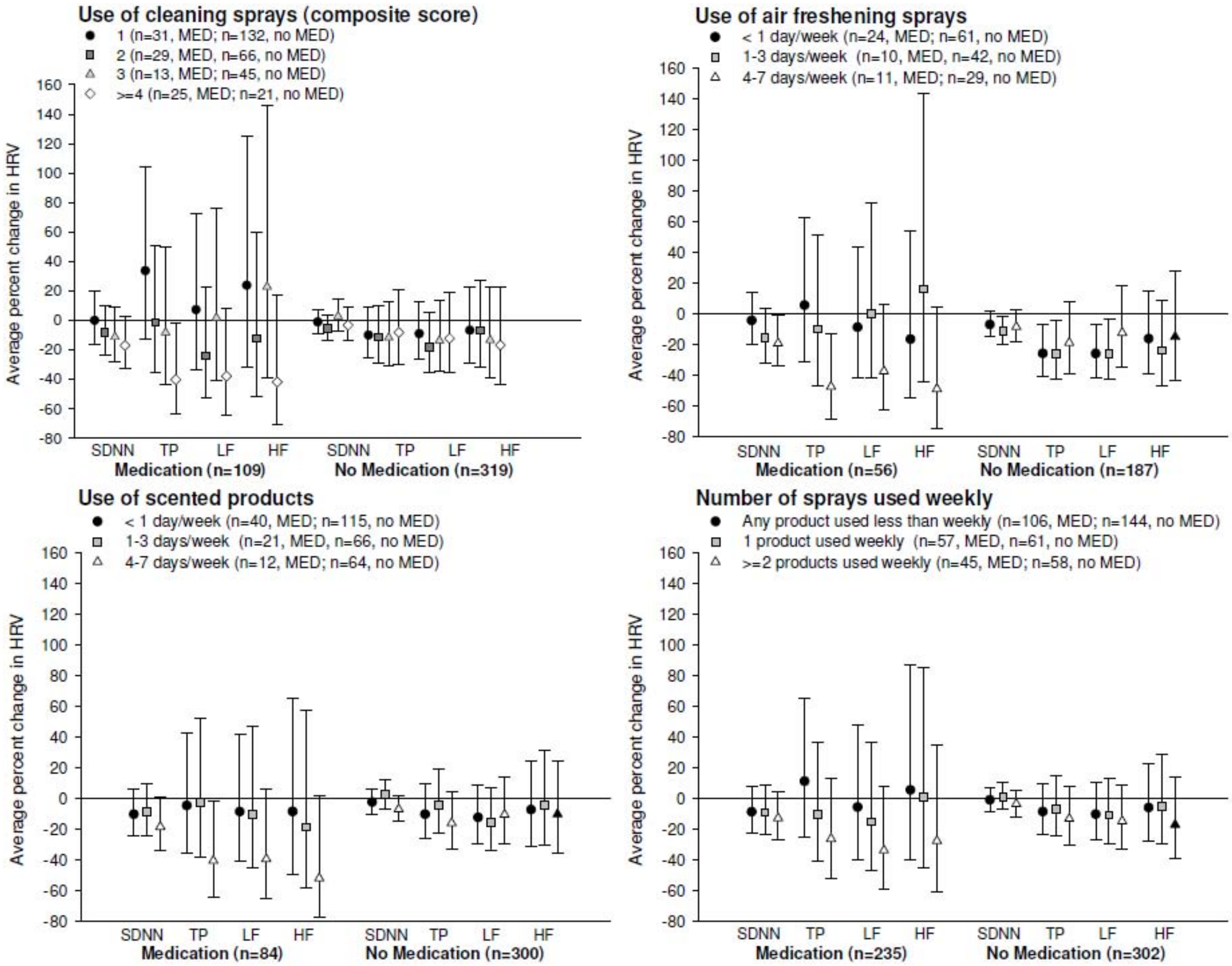

Supplemental Material, Figure 4 Adjusted average percent changes* in 24-hour SDNN, TP, LF, and HF in association with use of cleaning sprays, air freshening sprays, scented products, and with number of sprays used weekly after stratification by cardiovascular medication intake.

* 24-hr SDNN, TP, LF, and HF were modelled on the logarithmic scale in multiple linear regression as a function of each exposure in separate models and then transformed into average percent change relative to unexposed participants (n=11, medication (MED); n=23, no medication (NO MED)), after adjustment for gender, age, age squared, body mass index, body mass index squared, alcohol consumption, physical activity, smoking status, environmental tobacco smoke exposure, education, employment status, cardiovascular medication intake, uric acid levels, street and railway noise, traffic-related particulate matter, seasonal effects and study area.

Supplemental Material, Table 5 Adjusted average percent changes* in major indices of nighttime HRV in association with frequency use of household spray and scented products

	24-hour SDNN			Total power		
**Exposure variable**	**% change**	**95%CI**	**p-value for ordinal trend**	**% change**	**95%CI**	**p-value for ordinal trend**
Composite score variable for cleaning sprays						
Unexposed (n=66)		ref			ref	
1 (n=163)	-3.9	(-11.3, 4.1)	0.31	-2.0	(-17.3, 16.2)	0.27
2 (n=95)	-10.4	(-18.1, -2.0)		-13.1	(-28.1, 4.1)	
3 (n=58)	-3.9	(-13.1, 7.3)		1.0	(-18.9, 25.9)	
≥ 4 (n=46)	-4.9	(-15.6, 6.2)		-14.8	(-32.3, 8.3)	
Air freshening spray (days/week)						
Unexposed (n=66)		ref			ref	
<1 (n=85)	-9.5	(-18.1, 0.2)	0.17	-9.5	(-26.7, 10.2)	0.18
1-3 (n=52)	-10.4	(-20.5, 0.6)		-16.5	(-33.6, 5.9)	
4-7 (n=38)	-11.3	(-21.3, 1.2)		-13.9	(-33.6, 11.6)	
Scented products (days/week)						
Never (n=66)		ref			ref	
<1 (n=155)	-5.8	(-13.1, 3.0)	0.28	-3.0	(-18.1, 15.0)	0.20
1-3 (n=87)	-3.0	(-11.3, 6.2)		-5.8	(-22.1, 13.9)	
4-7 (n=76)	-6.8	(-15.6, 2.0)		-11.3	(-26.7, 8.3)	
Number of sprays used weekly						
Unexposed (n=66)		ref			ref	
Any spray < 1 day/week (n=250)	-4.9	(-12.2, 3.0)	0.54	-3.9	(-18.9, 12.7)	0.42
1 spray ≥ 1 day/week (n=118)	-2.0	(-10.4, 7.3)		-3.0	(-19.7, 17.4)	
≥ 2 sprays ≥ 1 day/week (n=103)	-5.8	(-13.9, 3.0)		-8.6	(-25.2, 10.5)	

* Each variable HRV metric was modeled on the logarithmic scale in multiple linear regression as a function of each exposure variable (continuous, or categorical) in separate models, and adjusted for gender, age, age squared, body mass index, body mass index squared, alcohol consumption, physical activity, smoking status, environmental tobacco smoke exposure, education, employment status, cardiac medication, uric acid levels, street and railway noise, seasonal effects and study area.

Supplemental Material, Table 6 Adjusted average percent changes* in major indices of HRV in association with frequent use household spray and scented products

Exposure variable	% change	95%CI	% change	95%CI	% change	95%CI	% change	95%CI
Composite score variable for cleaning sprays								
Never or ≤ 1 (n=139)		ref		ref		ref		ref
2 (n=95)	-5.2	(-11.1, 1.1)	-8.1	(-21.1, 7.0)	-16.5	(-29.5, -1.3)	-9.0	(-26.5, 12.5)
3 (n=58)	0.3	(-7.2, 8.3)	-9.5	(-24.6, 8.5)	-6.5	(-23.5, 14.4)	-6.0	(-27.1, 21.3)
≥ 4 (n=46)	-6.3	(-14.3, 2.4)	-18.2	(-33.8, 1.0)	-17.4	(-34.6, 4.4)	-25.3	(-44.4, 0.4)
Air freshening spray (days/week)								
Never or < 1 (n=151)		ref		ref		ref		ref
1-3 (n=52)	-8.7	(-15.6, -1.2)	-12.1	(-28.1, 7.4)	-8.1	(-25.3, 13.1)	-7.8	(-30.2, 22.0)
4-7 (n=38)	-7.8	(-15.8, 0.9)	-19.2	(-35.8, 1.7)	-7.7	(-27.2, 17.0)	-17.7	(-40.3, 13.2)
Scented products (days/week)								
Never or < 1 (n=221)		ref		ref		ref		ref
1-3 (n=87)	3.1	(-3.1, 9.7)	3.0	(-11.4, 19.7)	-6.7	(-20.9, 10.2)	-2.3	(-21.8, 22.0)
4-7 (n=76)	-6.2	(-12.3, 0.3)	-14.9	(-27.6, 0.1)	-8.4	(-23.4, 9.6)	-14.3	(-32.6, 9.0)
Number of sprays used weekly								
Never or any spray < 1 day/week (n=316)		ref		ref		ref		ref
1 spray ≥ 1 day/week (n=118)	1.0	(-4.4, 6.7)	-4.7	(-16.4, 8.6)	-4.6	(-17.7, 10.6)	-0.8	(-17.8, 19.8)
≥ 2 sprays ≥ 1 day/week (n=103)	-3.4	(-9.0, 2.5)	-13.7	(-25.2, -0.6)	-13.5	(-26.4, 1.6)	-17.9	(-33.1, 0.8)

* 24-hr SDNN , TP, LF, and HF were modeled on the logarithmic scale in multiple linear regression as a function of each exposure (discrete) in separate models and then transformed into average percent change relative. All effect estimates are relative to unexposed participants (n=66) in combination with participants who used specific product(s) of interest < 1 day/week, and are adjusted for gender, age, age^2, bmi, bmi^2, alcohol consumption, physical activity, smoking status, ETS exposure, education, employment status, cardiovascular medication intake, uric acid levels, street and railway noise, traffic-related PM_{10}, seasonal effects and study area.
